# Tranquilizer effect on the Lyapunov exponents of lame horses

**DOI:** 10.1016/j.heliyon.2020.e03726

**Published:** 2020-04-08

**Authors:** J. Zhao, D.B. Marghitu, J. Schumacher

**Affiliations:** a Department of Mechanical Engineering, 1418 Wiggins Hall, Auburn University, Auburn, AL, 36849, USA; b Department of Clinical Sciences, College of Veterinary Medicine, Auburn University, Auburn, AL, 36849, USA

**Keywords:** Bioengineering, Mechanical systems, Biomechanics, Biomechanical engineering, Animal behavior, Musculoskeletal system, Lyapunov exponent, Horse gait, Tranquilization, Nonlinear dynamics, Lameness examination

## Abstract

Tranquilization of horses with acepromazine has been used to suppress erratic head movements and increase the accuracy of a lameness examination. Some equine clinicians believe that tranquilization with acepromazine will make lameness more evident by causing the horse to focus on adjusting its gait to avoid limb pain rather than its surroundings. The aim of this study was to investigate the effect of acepromazine on the Lyapunov exponents of lame horses. Ten lame horses were trotted in a straight line for a minimum of 25 strides. Kinematic data created by head movement were analyzed. Nonlinear analysis methods were applied to lame horse locomotion. The effect of acepromazine on the largest Lyapunov exponents of the lame horses were investigated. There was no statistically significant effect of acepromazine on the maximum value of Lyapunov exponents. The nonlinear dynamic methods can be used to analyze the gait in horses. Local stability of horse gait remains unchanged after the administration of acepromazine.

## 1 Introduction

Lameness causes significant economic loss to the equine industry (USDA, 2001). When lameness is of moderate to severe intensity, subjective evaluation by a clinician experienced in lameness examination is sufficient for correctly identifying the lame limb and the results of diagnostic analgesia (Hinchcliff et al., 2013). However, when subjectively examining horses with subtle lameness, disparate diagnoses between equine clinicians were reported, indicating the need for objective examination in these cases (Keegan et al., 2010; McCracken et al., 2012). Body mounted inertial sensors have been widely used to objectively analyze gait in horses (Keegan et al., 2004, 2002; Rhodin et al., 2016). Inertial sensor systems have been found to provide accurate and repeatable data concerning identification of the lame limb and the intensity of lameness (Keegan et al., 2012; Sepulveda Caviedes et al., 2018). Good agreement between two commercial inertial sensor systems for lameness examination has been verified (Pfau et al., 2016). For unruly horses, tranquilization or sedation might be used to suppress erratic head movements and increase the reliability of the lameness examination (da Silva Azevedo et al., 2015). A kinematic study reported that even though sedation of horses with 0.01 mg/kg detomidine did not change their degree of lameness, their head height decreased significantly, whereas stride length, stride duration and stance duration increased significantly (Buchnner et al., 1999). Some equine clinicians believe that tranquilization with acepromazine will make lameness more evident because this drug has no analgesic properties (Pilsworth and Dyson, 2015), but other clinicians believe that acepromazine does not influence the degree of lameness (da Silva Azevedo et al., 2015; Taintor et al., 2016). In a study where acepromazine was administered to sound horses, significant reductions in speed, stride frequency, stride length and ground-to-lip distance were observed even though the coordination parameters, including regularity, symmetry, and stability of gait did not change (López-Sanromán et al., 2015). A better understanding of how acepromazine affects the gait of horses is needed to determine if this treatment will interfere with the interpretation of a lameness examination performed using an inertial sensor system.

Animal locomotion is a result of complex interactions among musculoskeletal system, sensory systems and the environment (Biewener and Daniel, 2010). While gait always follows a regular pattern, animal locomotion is actually nonperiodic with small and subtle stride-to-stride variations (Goshvarpour and Goshvarpour, 2012). Nonperiodic locomotion can be quasi-periodic, deterministic chaotic, or stochastic. Chaos, indicating sensitivity to initial conditions and long-term unpredictability, commonly exists in the activity of many biological structures such as the heart and brain (Williams, 1997). The natural stride-to-stride variation in nominal periodic locomotion is clearly distinguished from a random noise and contains valuable information about the generation of the locomotion pattern (Dingwell and Cusumano, 2000). In other words, each stride of locomotion is generated dependently of the past and future strides (Dingwell et al., 2001). Therefore, any method to examine locomotion that has the ability to characterize the inherently dynamic nature of the locomotion system is important.

The analyses of nonlinear dynamic phenomena in physiological systems play an important role in diagnosing the abnormal activities and predicting their behavior. Recurrence quantification analysis and central tendency measure have been used to compare differences in the complexity and variability of center of pressure in patients with knee osteoarthritis and healthy people (Negahban et al., 2016). Complexity loss and variability increase in centers of pressure were observed in patients with osteoarthritis of the knee compared with healthy controls. Sample entropy, adaptive fractal analysis and recurrence quantification analysis which quantifies the complexity, fractal properties and determinism, respectively, have been introduced to examine differences between walking overground or on a treadmill (Hollman et al., 2016). In that study, stride time complexity and self-similarity in stride time and stride length were reduced on the treadmill.

As a quantitative analysis method, nonlinear time series analysis has been introduced to quantify the sensitivity of the locomotor system to local perturbations that occur naturally during normal locomotion. When dynamic stability of older and younger human adults walking at the same percentage of preferable speeds were compared, older adults exhibited greater local instability than younger adults (Buzzi et al., 2003; Kang and Dingwell, 2008). However, gait instability is distinguished from gait variability given that increased local stability accompanied by increased gait variability was found in both young and elder human adults when walking speeds were decreased (Kang and Dingwell, 2008). The similar effect of walking speed on local stability was found in patients with neuropathic disease, indicating that the neuromuscular control system adopts a lower speed as a strategy to improve local stability (Dingwell et al., 2000; Dingwell and Cusumano, 2000). The global stability, on the other hand, quantifies the locomotor system's ability to resist larger perturbations (Dingwell et al., 2000). Studying nonlinear dynamic stability of human locomotion is of great importance for a better understanding of robotic systems (Tarnita and Marghitu, 2013; Yang and Wu, 2006) and has developmental implications for rehabilitation of people with an abnormal gait (Nessler et al., 2009). Local dynamic stability of gait during the transition between walk and run was found to be associated with long range correlations of stride interval (Jordan et al., 2009).

Nonlinear analyses of kinematic measurements have been applied to study canine gait. Gait stability of normal and arthritic greyhounds was compared using the Floquet theory with the results indicating that the arthritic dogs had a less stable gait than that of the normal dogs (Marghitu and Nalluri, 1997). The dynamic interaction of English Pointer dogs and the bearing surface has been analyzed by calculating the coefficients of restitution (Marghitu et al., 2003). Significant differences in coefficients of restitution among paw pads were found, with the metapodial pads consistently found to be the least plastic. The walking and pacing stability of German Shepherd dogs have been investigated using Lyapunov exponents with the results suggesting that stability decreased when speed increased (Tian et al., 2011).

Previous studies of horse gait focused only on the linear kinematic and kinetic measures, such as means and standard deviations of speed, stride length, and propulsive power. Given evidence that nonlinear analysis methods provided valuable insight for evaluating gait for other species (Fallah Yakhdani et al., 2010; Marghitu and Nalluri, 1997), Non-linear analysis methods with the ability to characterize the intrinsic property of the locomotor system should be considered to supplement the analysis of horse gait. The purpose of this study was to assess the effect of tranquilization of horses with acepromazine on the lameness examination, and to quantify acepromazine's effect on local dynamic stability of gait in lame horses.

## 2 Materials and methods

### 2.1 Study design

Ten horses from the equine teaching herd of the College of Veterinary Medicine at Auburn University were enrolled in the study after they were determined to be lame on a forelimb by subjective evaluation of head carriage by one of the investigators (J.S.), experienced at subjective evaluation of lame horses. These horses had ranged in weight from 409 to 510 kg and had average weight of 468.9 kg. These horses were then objectively evaluated using an inertial sensor system (Lameness Locator; Equinosis, LLC, Columbia, MO, USA) that identifies asymmetry of gait at a trot. This inertial sensor system provides appropriate accuracy and sensitivity for clinical use (Keegan et al., 2012, 2011). The inertial sensor system recorded differences in maximum and minimum head height (HDmax and HDmin, respectively). For each trial, HDmax and HDmin were evaluated to determine if the threshold value of lameness was reached. The threshold value for forelimb lameness was ±6 mm for HDmax and HDmin. The overall amplitude of forelimb lameness for all strides was calculated as vector sum (VS) of HDmax and HDmin and has a threshold of 8.5. The inertial sensor system verified that all horses were lame on a forelimb. Using a randomized two-way crossover design, the horses were randomly split into two groups: (a) 5 horses were first administered 10 mg of acepromazine (PromAce, Boehringer Ingleheim Vetmedica, Inc, St. Joseph, MO) intravenously before being objectively evaluated and at another time receiving no treatment before being similarly evaluated, whilst (b) the other 5 horses received no treatment before being objectively evaluated and then at another time receiving 10 mg of acepromazine administered intravenously before being similarly evaluated (Taintor et al., 2016). Each horse was trotted every 5 min for 45 min after it was administered acepromazine or no treatment. After each treatment, horses were returned to their permanent pasture for five days until their next treatment. For consistency of speed, the same handler trotted all horses. This study was approved by the university's Animal Care and Use Committee (Protocol Number, 2013–2355).

### 2.2 Kinematic data

**Figure 1:**
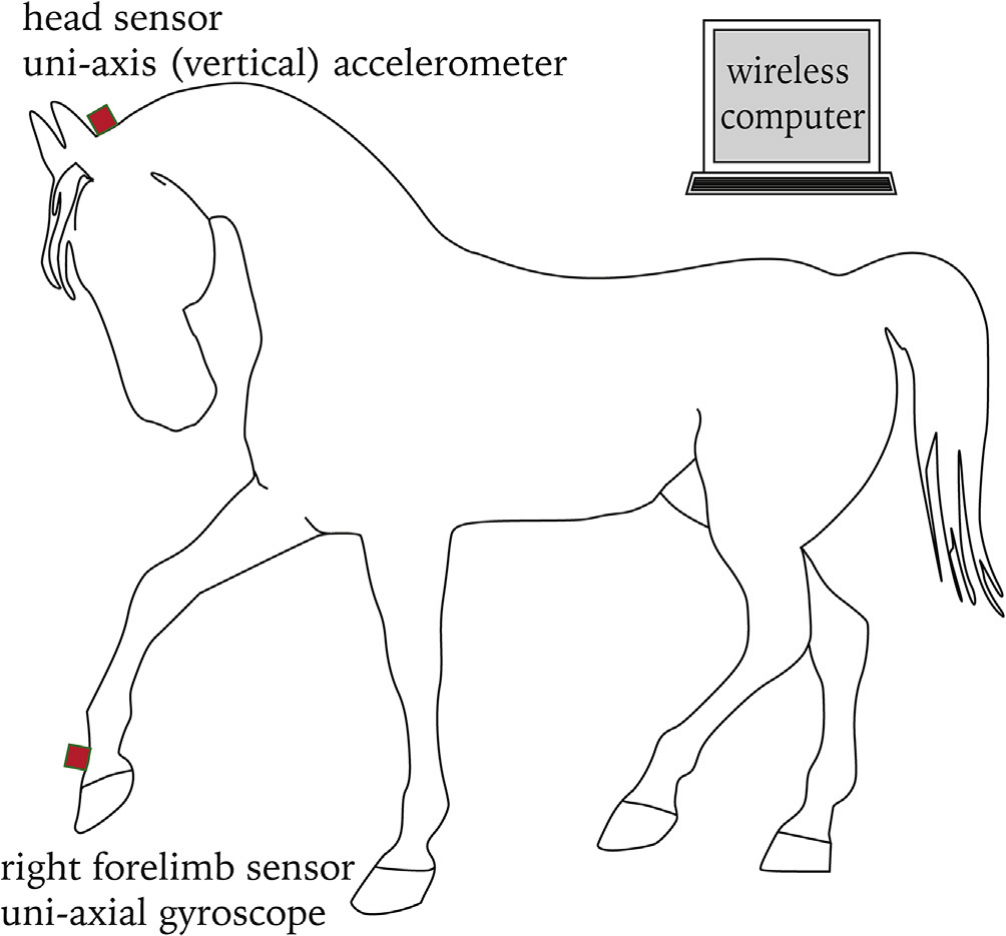
Inertial sensors attached to the horse.

**Figure 2:**
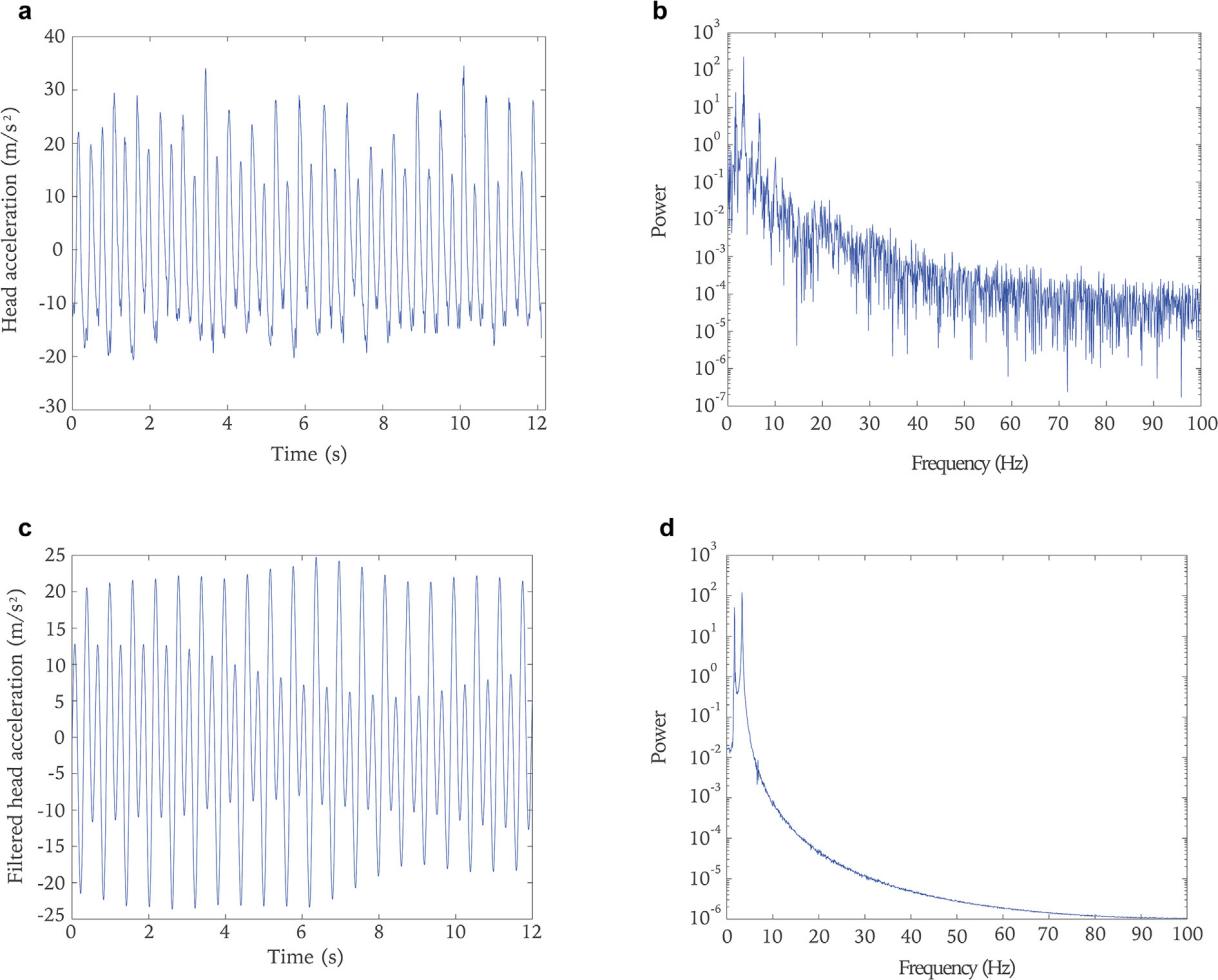
a. Twenty strides of head acceleration data from a lame horse during trotting. Data were sampled at 200 Hz in real time. b. The Fourier power spectrum of the time series shown in a. The continuous and broadband spectrum indicates a nonperiodic behavior. c. The filtered data of the signal in a. The nonperiodic movements have been excluded from the original signal. d. The Fourier power spectrum for the filtered data in c. The fundamental wave is located at 1.654 Hz and the first harmonic is located at 3.309 Hz. A considerably high amplitude of the fundamental wave indicates a lame forelimb.

Each horse was trotted in a straight line for a minimum of 25 strides while equipped with two wireless inertial sensors as shown in Figure 1. A single-axis accelerometer transducer which measures acceleration along the vertical (dorsoventral) axes of the horse was attached to the head halter with a Velcro patch. A gyroscopic transducer which measures the angular velocity of the foot was attached to the dorsal surface of the pastern of the right forelimb using a wrap provided by the company. The gyroscopic transducer was used to determine the onset and end of each stride cycle. Data were collected from each of those trotting trials. The collected data were transmitted at 200 Hz to a computer in real time using Bluetooth wireless technology. All the horses were familiar with the experimental environment. Data from a total of 18 trials were collected for each horse. Head acceleration data gathered for twenty strides were extracted from each trial for further analysis as shown in Figure 2a.

First, the time series was analyzed. Knowing whether a time series is periodic provides important information about how to proceed in a nonlinear analysis. In order to reveal periodicity of the analyzed data, a Fast Fourier Transform algorithm based on the idea that any signal can be decomposed into constituent sinusoidal waves was implemented to the time series:

(1) $$y=a_{0}+\sum_{i=1}^{n}a_{i}\;\cos\left(i\omega x\right)+b_{i}\;\sin\left(i\omega x\right)$$

where $a_{0}$ models a constant term in the data and is associated with the *i* = 0 cosine term; *ω* is the fundamental frequency of the signal; *n* is the number of terms in the series. A power spectrum composed of an infinite number of sinusoids with continuous frequencies was obtained from the transform. Patterns from power spectrum was used to detect periodicity. Periodic signals show peaks at its fundamental and harmonic frequencies. For quasi-periodic signals, peaks are given at linear combinations of two or more incommensurable frequencies. In the cases of chaotic or random signals, broadband spectra is exhibited (Sornsanam and Sukawat, 2012). The power spectrum with a broadband spectra shown in Figure 2b suggests that the time series is not periodic. A filtering method which excludes the nonperiodic parts of the collected signal has been applied (Peham et al., 1996). The filtered data in the time domain are represented in Figure 2c. From the power spectrum of the filtered signal, the fundamental wave was found to be located at 1.654 Hz and the first harmonic was located at 3.309 Hz as depicted in Figure 2d. After filtering, a considerably high amplitude of the fundamental wave indicates lameness in a forelimb. Motion symmetry was calculated using the following equation:

(2) $$symmetry\left(\%\right)=\frac{AFHW}{AFW+AFHW}*100$$

where AFW is the amplitude of the fundamental wave, and AFHW is the amplitude of the first harmonic wave. The greater the ratio of the amplitude of the fundamental wave to that of the first harmonic wave, the more severe the lameness.

**Figure 3:**
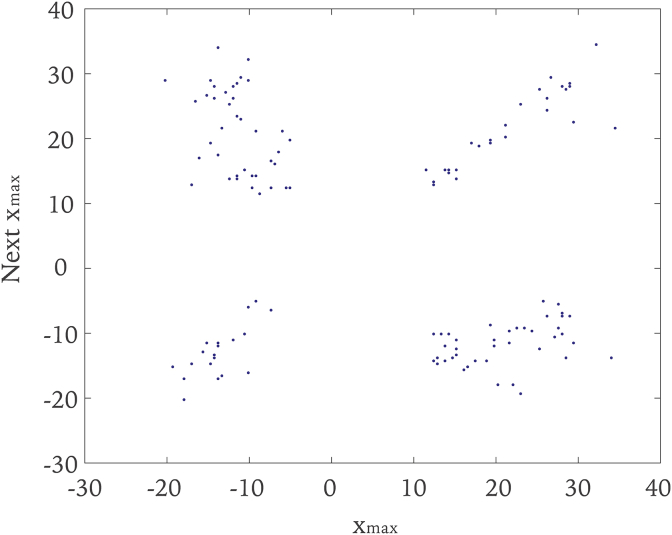
The Poincaré map associated with the time series in Figure 2a. The scatter of dots indicates that the time series is not periodic or quasiperiodic.

The Poincaré map provided another view of the periodicity of the system. The Poincaré map is the cross section of a trajectory in state space with a lower-dimensional subspace which is transverse to the flow (Strogatz, 2018). It works as a function that finds the point where the trajectory will return to at that section, given the position of the previous intersection at the selected section. The Poincaré map helps to identify the type of the attractor. For a periodic attractor, the Poincaré map consist of a finite set of points with the number of points corresponds to the period of the attractor. If a system has quasiperiodic motion, the Poincaré map will be a closed orbit. The Poincaré map of a chaotic attractor appears as an infinite number of points. The Poincaré map with a scatter of dots shown in Figure 3 indicates that the time series was not periodic or quasiperiodic. In Figure 3, the maximum values of the head acceleration data were found and plotted with each value on the abscissa and its successor on the ordinate.

### 2.3 State space reconstruction

Based on the result from the above analysis, the second step for the nonlinear analyses was to reconstruct the state space *S* from the original time series by using the method of delays (Packard et al., 1980):

(3) X(t)=[x(t),x(t+T),x(t+2T),...,x(t+(m−1)T)]

where *X*(*t*) is the reconstructed state of the system at time *t*, *x*(*t*) is the collected head acceleration data, *T* is the time delay, and *m* is the embedding dimension. Collected head acceleration data was a projection of the state space of the observed system. The purpose of the delays embedding method was to unfold this projection back to the state space that represents the system (Kennel et al., 1992). The reconstructed state of the system preserves the same dynamical properties as the original system.

**Figure 4:**
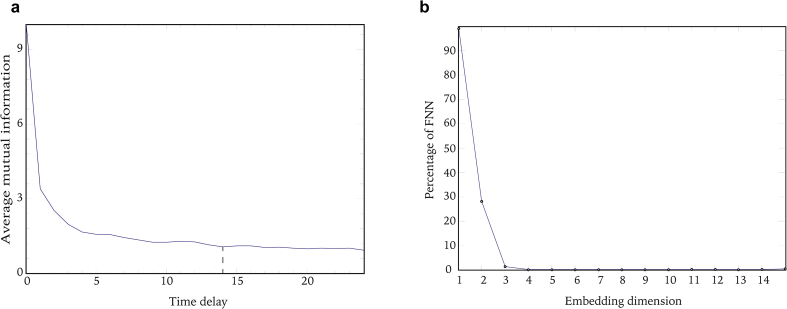
a. The average mutual information for the time series in Figure 2a. The first minimum of the average mutual information is attained when the time delay T = 14. b. The percentage of false nearest neighbors for the time series shown in Figure 2a using time delay T = 14. The embedding dimension m = 4 was chosen where the number of false nearest neighbors dropped to zero.

The proper time delay is needed to ensure that the delayed coordinates are independent from the original time series. Average mutual information, quantifying the amount of information shared between the delayed time series and the collected head acceleration data, decreases with the increase of time delay. The proper time delay *T* is chosen at where the first minimum of average mutual information occurs (Fraser and Swinney, 1986). The average mutual information as a function of time delay is shown in Figure 4a. The first minimum is attained when the time delay is 14.

The appropriate embedding dimension *m* is determined from the false nearest neighbors (FNN) method (Kennel et al., 1992). The dimension of a system is the number of independent variables needed to specify the system (Fraser and Swinney, 1986). A false neighbor is a point that is a false neighbor only in a lower embedding dimension and will become a true neighbor as the embedding dimension increased to a higher enough value. The number of false nearest neighbors declines with increasing embedding dimension and *m* is selected when it drops to zero. Selecting the minimum effective embedding dimension is crucial for saving the computation time and reducing the noise effect. The percentage of false nearest neighbors versus embedding dimension associated with the time series in Figure 2a is shown in Figure 4b. It was observed that the number of false nearest neighbors was zero when the embedding dimension was 4 for the time series. The embedding dimension for all the trials of this study were calculated as 3, 4, 5.

### 2.4 Lyapunov exponents

The values of the Lyapunov exponents, which measure the rates of separation of nearby trajectories along dominant directions in state space, are quantitative measures of chaotic behaviors and characterize the local stability of the system (Kantz and Schreiber, 2003). Positive exponents indicate local instability with the larger number suggesting greater sensitivity to the initial conditions of the system. A periodic system possesses all the Lyapunov exponents as negative or zero, while a chaotic system has one or more positive Lyapunov exponents (Wolf et al., 1985).

Calculating the entire Lyapunov exponents spectrum is complicated and unnecessary for real world data. It is expected that the nearby trajectories will diverge, on average, at an exponential rate given by the largest Lyapunov exponent since the exponential divergence in this direction quickly dominate separation along the other directions (Rosenstein et al., 1993). The average divergence of the *j*^th^ pair of nearest neighbors in state space is described by an exponential function:

(4) $$d_{j}\left(i\right)=C_{j}e^{\lambda_{1}\left(i\Delta t\right)}$$

where *C*_j_ is the initial separation, *λ*_1_ is the largest Lyapunov exponent. By taking the logarithm of both sides of Eq. (2), the logarithmic separation was obtained.

Therefore, the largest Lyapunov exponent was estimated as the slope of the best-fit line of the logarithmic separation, expressed by

(5) $$y\left(i\right)=\frac{\left\langle \ln\left(d_{j}\left(i\right)\right)\right\rangle }{\Delta t}$$

where < > denotes the average of all values of *j*.

**Figure 5:**
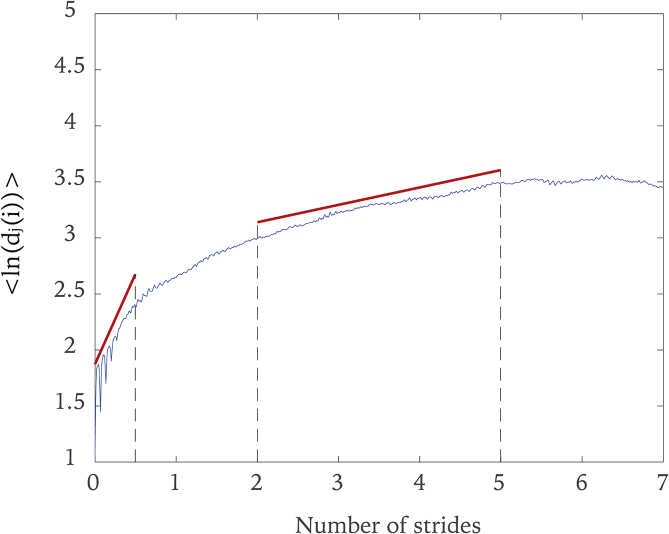
The average logarithmic divergence of neighboring trajectories. The short-term and long-term largest Lyapunov exponents are calculated as the slopes of the curves between 0-0.5 stride and between 2-5 strides.

Short-term and long-term exponents can be calculated over two different scaling regions as estimates of the maximum finite time Lyapunov exponents (Dingwell et al., 2001). The average logarithmic divergence of neighboring trajectories of a time series in this study is shown in Figure 5. For our study, short-term exponents were calculated from the best-fit linear slopes of the local divergence curves between 0 and 0.5 stride and long-term exponents were computed between 2 and 5 strides, as indicated by the slopes of two lines in Figure 5.

### 2.5 Statistical analysis

Statistical analyses were performed using the statistical software SAS (SAS Studio 3.8, SAS Institute Inc., Cary, NC, USA.). The fundamental frequency and motion symmetry data were subjected to a reciprocal and logit transformation, respectively, to approximate a normal distribution. Linear mixed effects model Y=Xβ+Zμ+e were performed to determine if acepromazine had an effect on each variable (fundamental frequency, motion symmetry, short-term and long-term largest Lyapunov exponents), where Y was the vector of the observations, X was the treatment design matrix (treatment with acepromazine or no treatment; data collection at 5, 10, 15, 20, 25, 30, 35, 40, 45 min), β was the vector of fixed treatment effects, Z was the subject design matrix (horse), μ was a vector of random subject-specific effects, e was a vector of random experimental error. To account for the correlation and variability among repeated measures on the same horse, five correlation structures including first-order autoregressive, compound symmetry, unstructured, Toeplitz and variance components have been tested and compared using the Akaike's information criteria (Kincaid, 2005). The Kenward-Roger correction has been applied to all the models (Littell et al., 2006). Values of *P ≤* 0*.*05 were considered to be significantly different.

## 3 Results

Data generated by the inertial sensor system indicated that all horses had a forelimb lameness. The mean and median values of VS with standard deviations (SD) are shown for horses administered acepromazine or no treatment in Table 1 (Taintor et al., 2016).

**Table 1:** Mean, standard deviations (SD) and median values of vector sum (VS) between horses after treatment with acepromazine or no treatment.

Time after treatment (min)	VS after treatment with acepromazine			VS after no treatment		
	Mean	SD	Median	Mean	SD	Median
5	17.13	11.44	15.91	16.26	12.19	10.77
10	17.28	10.90	17.25	19.69	14.53	13.96
15	19.02	11.80	12.97	23.29	15.04	20.87
20	20.20	10.35	16.21	23.01	11.45	19.28
25	19.36	13.24	15.06	21.60	12.36	21.11
30	18.89	11.03	16.74	20.57	9.11	20.09
35	19.18	12.08	14.89	21.75	8.55	22.31
40	18.77	11.47	16.42	19.97	10.69	20.13
45	17.92	9.47	16.37	23.65	12.43	19.54

**Figure 6:**
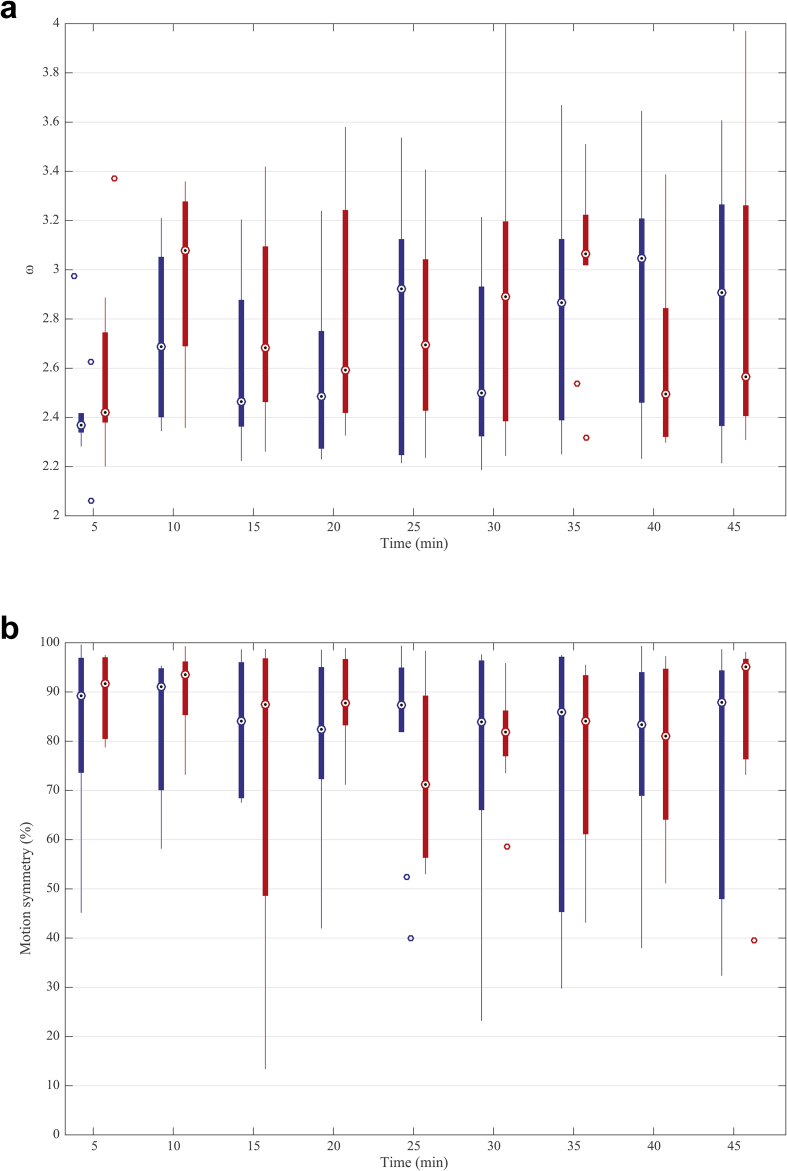
a. Boxplot of the fundamental frequencies of head acceleration data from ten horses at 5, 10 and every 5 min over a total period of 45 min after treatment with acepromazine (blue) and no treatment (red). b. Boxplot of the motion symmetry of ten horses at 5, 10 and every 5 min over a total period of 45 min after treatment with acepromazine (blue) and no treatment (red). Dotted circles within boxes illustrate the median values. The edges of the boxes are the 25th and 75th percentiles. Whickers indicate the most extreme data points not considered outliers. Circles outside boxes indicate the outliers.

The unstructured correlation structure provided the best fit for all the variables based on the Akaike's information criteria. The fundamental frequency and motion symmetry of data from ten horses are shown in Figure 6a, b. Treatment with acepromazine had a significant effect on the fundamental frequency (P = 0.0175). The fundamental frequency was significantly lower after treatment with acepromazine compared with no treatment. Times of evaluation were not significantly different (P = 0.1125). With regard to the motion symmetry, no significant differences were found after treatment with acepromazine or at different time of evaluation (P = 0.8302 for treatment, P = 0.1472 for time).

**Figure 7:**
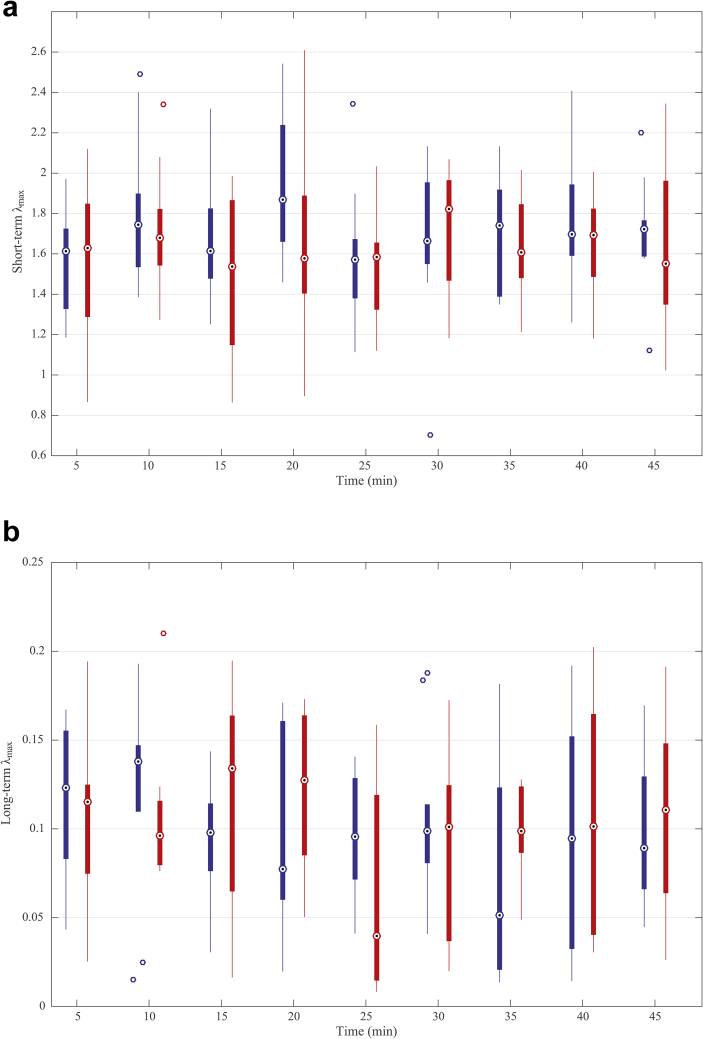
a. Boxplot of the short-term largest Lyapunov exponents of head acceleration data from ten horses at 5, 10 and every 5 min over a total period of 45 min after treatment with acepromazine (blue) and no treatment (red). b. Boxplot of the long-term largest Lyapunov exponents of head acceleration data from ten horses at 5, 10 and every 5 min over a total period of 45 min after treatment with acepromazine (blue) and no treatment (red). Dotted circles within boxes illustrate the median values. The edges of the boxes are the 25th and 75th percentiles. Whickers indicate the most extreme data points not considered outliers. Circles outside boxes indicate the outliers.

The short-term and long-term largest Lyapunov exponents of head acceleration data from ten horses are presented in Figure 7a, b. All the values were positive. Values of the short-term and long-term exponents varied from 0.7 to 2.6 and 0.008 to 0.21, respectively. Median values of the short-term and long-term exponents were distributed in the region between 1.53 to 1.87 and 0.04 to 0.14, respectively. For short-term largest Lyapunov exponents, no significant differences were found after treatment with acepromazine compared with no treatment (P = 0.5718). Times of evaluation were not significantly different (P = 0.6133). Similar results were obtained for long-term largest Lyapunov exponents (P = 0.6919 for treatment, P = 0.2208 for time). Results imply that the sensitivity of the system to infinitesimally small perturbations was not changed after treatment.

## 4 Discussion

Based on results of this study, the intravenous administration of 10 mg acepromazine to lame horses did not change the largest Lyapunov exponents and motion symmetry. The results suggest that administration of acepromazine for lameness evaluation would not affect the local dynamic stability of horse gait. A pharmacokinetics and pharmacodynamics study of intravenously administered acepromazine in horses found that the central nervous system effects peaked at 20 min after administration (Marroum et al., 1994). We did not observe any significant change in the largest Lyapunov exponents or motion symmetry at times of evaluation. Our results are in good agreement with the results of other studies which evaluated lameness of horses before and after administration of acepromazine using the same inertial sensor based motion analysis system (da Silva Azevedo et al., 2015; Taintor et al., 2016). The administration of acepromazine to improve control of unruly horses for lameness evaluation is, therefore, unlikely to influence the results of a lameness examination conducted using an inertial, sensor based motion analysis system.

**Figure 8:**
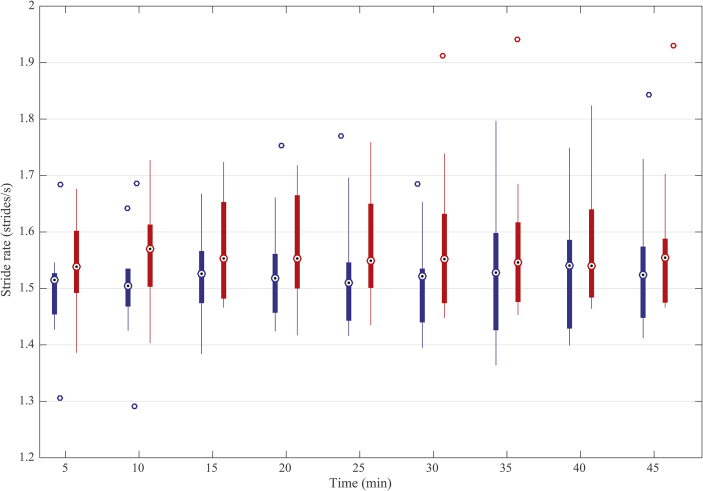
Boxplot of the stride rate of ten horses at 5, 10 and every 5 min over a total period of 45 min after treatment with acepromazine (blue) and no treatment (red). Dotted circles within boxes illustrate the median values. The edges of the boxes are the 25th and 75th percentiles. Whickers indicate the most extreme data points not considered outliers. Circles outside boxes indicate the outliers.

In this study horses were trotted in hand by a single handler and velocity was not directly quantified. However, the inertial sensor system, which calculates stride rate, may be used to indirectly monitor velocity because stride rate has been shown to increase linearly with increased velocity (Robert et al., 2002). Stride rates of the ten horses are shown in Figure 8. There was no significant difference in the stride rate after treatment with acepromazine compared with no treatment or at different time of evaluation (P = 0.1969 for treatment, P = 0.079 for time). Even if the velocity may have varied slightly, one study showed no significant differences in forelimb kinematics (HDmax, HDmin, VS) when velocity of the trot on a treadmill was varied (Moorman et al., 2017).

Administering acepromazine before a lameness examination can reduce the occurrence of erratic head movement, which will improve data collection (da Silva Azevedo et al., 2015). Based on this observation we assumed that it would be easier to observe the effect of acepromazine by evaluating head movement. Therefore, for this study, the head movements were used to calculate the largest Lyapunov exponents. Likewise, nonlinear analysis method can be applied to the pelvic movement to assess the effect of tranquilization on the hindlimb.

There are some limitations of this study. The variability of trial-to-trial largest Lyapunov exponent is still large. In regard to the calculation of the largest Lyapunov exponent, there is no consensus on which time scale should be used (Reynard and Terrier, 2014). Further studies are needed to improve the reliability of this method. Given evidence that slightly but significantly greater largest Lyapunov exponents were found in human patients with peripheral neuropathy compared with healthy controls (Dingwell and Cusumano, 2000), we speculate that the largest Lyapunov exponent might increase after the administration of acepromazine if the horses were more lame than the horses we evaluated. However, further studies comparing the gait from sound and lame horses with different lameness intensity are required to assess the clinical applicability of this method of gait evaluation.

## 5 Conclusions

A key finding in this study is that even though tranquilization with acepromazine altered the locomotion pattern of lame horses as indicated by the fundamental frequency, the local dynamic stability and motion symmetry were not affected. The nonlinear dynamic methods can be used to analyze the gait in horses, but more research is needed to improve the intra/inter horse reproducibility.

## Declarations

### Author contribution statement

J. Zhao, D. B. Marghitu: Conceived and designed the experiments; Analyzed and interpreted the data; Contributed reagents, materials, analysis tools or data; Wrote the paper.

J. Schumacher: Conceived and designed the experiments; Performed the experiments; Contributed reagents, materials, analysis tools or data; Wrote the paper.

### Funding statement

This research did not receive any specific grant from funding agencies in the public, commercial, or not-for-profit sectors.

### Competing interest statement

The authors declare no conflict of interest.

### Additional information

No additional information is available for this paper.
